# Breathing 100% oxygen during water immersion improves postimmersion cardiovascular responses to orthostatic stress

**DOI:** 10.14814/phy2.13031

**Published:** 2016-12-16

**Authors:** John P. Florian, Ki H. Chon, Luca Faes, Barbara E. Shykoff

**Affiliations:** ^1^ Navy Experimental Diving UnitPanama CityFlorida; ^2^ Department of Biomedical EngineeringUniversity of ConnecticutStorrsConnecticut; ^3^ Bruno Kessler FoundationTrentoItaly; ^4^ BIOtechUniversity of TrentoTrentoItaly

**Keywords:** Autonomic nervous system, blood flow, blood pressure, heart rate variability, hyperoxia, orthostatic tolerance, water immersion

## Abstract

Physiological compensation to postural stress is weakened after long‐duration water immersion (WI), thus predisposing individuals to orthostatic intolerance. This study was conducted to compare hemodynamic responses to postural stress following exposure to WI alone (Air WI), hyperbaric oxygen alone in a hyperbaric chamber (O_2_ HC), and WI combined with hyperbaric oxygen (O_2_ WI), all at a depth of 1.35 ATA, and to determine whether hyperbaric oxygen is protective of orthostatic tolerance. Thirty‐two healthy men underwent up to 15 min of 70° head‐up tilt (HUT) testing before and after a single 6‐h resting exposure to Air WI (*N* = 10), O_2_ HC (*N* = 12), or O_2_ WI (*N* = 10). Heart rate (HR), blood pressure (BP), cardiac output (Q), stroke volume (SV), forearm blood flow (FBF), and systemic and forearm vascular resistance (SVR and FVR) were measured. Although all subjects completed HUT before Air WI, three subjects reached presyncope after Air WI exposure at 10.4, 9.4, and 6.9 min. HUT time did not change after O_2_ WI or O_2_ HC exposures. Compared to preexposure responses, HR increased (+10 and +17%) and systolic BP (−13 and −8%), and SV (−16 and −23%) decreased during HUT after Air WI and O_2_ WI, respectively. In contrast, HR and SV did not change, and systolic (+5%) and diastolic BP (+10%) increased after O_2_ HC. Q decreased (−13 and −7%) and SVR increased (+12 and +20%) after O_2_ WI and O_2_ HC, respectively, whereas SVR decreased (−9%) after Air WI. Opposite patterns were evident following Air WI and O_2_ HC for FBF (−26 and +52%) and FVR (+28 and −30%). Therefore, breathing hyperbaric oxygen during WI may enhance post‐WI cardiovascular compensatory responses to orthostatic stress.

## Introduction

Physiological compensation to postural stress is altered after long‐duration water immersion (WI), thus predisposing individuals to blood pressure instability and orthostatic intolerance (Stegemann et al. 1975; Faes et al. 2013; Florian et al. 2013). During WI, central blood volume increases due to fluid shifts from the intracellular and interstitial fluid compartments to extracellular compartment (Epstein 1992) and redistribution of blood volume from the lower extremities to the thorax (Greenleaf et al. 1983; Greenleaf 1984; Pendergast et al. 2015). Consequently, there is a shift toward increased parasympathetic and decreased sympathetic tone (Mano et al. 1991; Tripathi 2011; Florian et al. 2013), together with augmented excretion of fluid and electrolytes (Norsk et al. 1985; Epstein 1992). After egress from the water, plasma volume is reduced by 10–15% (Johansen et al. 1992; Boussuges et al. 2009; Florian et al. 2013), and with postural stress, the resulting deterioration of baroreflex sensitivity/coupling (Faes et al. 2013) and an inability to augment systemic vascular resistance may contribute to post‐WI orthostatic intolerance (Iwase et al. 2000; Florian et al. 2013).

Exposure to high partial pressures of inspired oxygen (hyperoxia) may, through direct and indirect effects on the vasculature and autonomic nervous system, augment post‐WI cardiovascular compensatory responses to orthostatic stress. Acute exposure to hyperoxia increases systemic vascular resistance via a local (nonsympathetic) mechanism (Seals and Victor 1991; Lund et al. 1999) and decreases resting heart rate (HR), stroke volume (SV), cardiac output (Q), and regional blood flow (Torbati et al. 1979; Gole et al. 2011). High‐frequency HR variability (HF_HRV_) is augmented and the low‐frequency/high‐frequency ratio (LF/HF_HRV_) is diminished with increasing partial pressure of O_2_ (Lund et al. 1999, 2003; Shibata et al. 2005). The change in cardiac regulation may be baroreflex‐mediated (Shibata et al. 2005; Demchenko et al. 2013) or due to reductions in cardiac sympathetic activity through unloading of chemoreceptor tonic reflex drive (Fukuda et al. 1989).

Although several studies have shown that vascular and cardiac changes may persist for a short duration after hyperoxia (Torbati et al. 1979; Gole et al. 2011), the physiological effects and impact of long‐duration hyperoxia on post‐WI orthostatic responses have not been evaluated. Therefore, the purpose of this study was to compare hemodynamic responses to postural stress following WI alone (Air WI), hyperbaric oxygen alone in a hyperbaric chamber (O_2_ HC), and WI combined with hyperbaric oxygen (O_2_ WI). Previously published data (Florian et al. 2013) from the Air WI phase are included in this manuscript for comparison purposes. WI was at the bottom a 15‐ft pool, with the lung centroid at approximately 12 ft (1.35 atmospheres absolute [ATA]), and pressure was matched during O_2_ HC. During supine rest and 15 min of 70° head‐up tilt (HUT), we measured hemodynamic variables, spontaneous baroreflex sensitivity, and time‐ and frequency‐domain measures of HRV. We hypothesized that cardiovascular stability during orthostasis would be diminished after a 6‐h Air WI, but that breathing 100% O_2_ during a 6‐h WI would enhance post‐WI orthostatic cardiovascular compensatory responses through blood volume protective mechanisms during WI and sustained vasoconstriction after WI.

## Methods

### Subjects

Thirty‐two healthy men (10 Air WI, 10 O_2_ WI, and 12 O_2_ HC), whose physical characteristics at screening are presented in Table 1, participated in the study. All participants were experienced military divers with an average of 9 years diving experience. They were healthy, active, normotensive nonsmokers who were not taking any medications that would affect responses in the study. Each subject underwent medical screening that included complete blood count, complete metabolic panel, lipid profile evaluation, urinalysis, physical examination, skinfold body fat measurement, and determination of maximal oxygen uptake ($\dot{V}$O_2 max_). Approval was obtained from the Institutional Review Board of the Navy Experimental Diving Unit. Each subject gave written informed consent, and all procedures conformed to the Declaration of Helsinki.

**Table 1:** Subject characteristics

	Air WI (*n* = 10)	O_2_ WI (*n* = 10)	O_2_ HC (*n* = 12)
Age, years	34 ± 10 (19–44)	34 ± 6 (28–44)	27 ± 5 (20–36)
Height, cm	179 ± 6	179 ± 6	179 ± 6
Weight, kg	85 ± 7	88 ± 7	86 ± 8
BMI, kg/m^2^	26 ± 1	27 ± 2	27 ± 2
Body fat, %	19 ± 4	18 ± 3	14 ± 4
*V*O_2 max_, mL/kg/min	53 ± 10	55 ± 10	47 ± 5
SBP, mmHg	124 ± 8	124 ± 10	124 ± 13
DBP, mmHg	76 ± 6	77 ± 7	76 ± 10
Hemoglobin, mg/dL	15 ± 1	15 ± 1	14 ± 1
Hematocrit, %	44 ± 3	44 ± 2	42 ± 2

Values are mean ± SD, with range in parentheses.

WI, water immersion; HC, hyperbaric chamber; SBP, systolic blood pressure; DBP, diastolic blood pressure.

### Study design

Subjects abstained from alcohol for 2 days, caffeine and strenuous exercise for 1 day, and food and drink (except water) for 2 h before reporting to the laboratory in the morning. Subjects wore running shorts and T‐shirts for all visits. Each subject underwent physiological testing before and after a 6‐h WI or HC trial. All physiological testing was completed in a laboratory (air temperature 22–24°C) adjacent to the immersion tank and HC. After completing pre‐WI or ‐HC testing (see Protocol for pre‐ and posttrial testing), each subject received a standardized snack, submitted a urine sample for measurement of urine specific gravity, emptied his bladder, and was weighed. For WI trials, a condom catheter was applied to collect urine during the dive. Subsequently, for WI trials each subject was immersed in the tank, surfaced after the third hour for a 10‐min lunch break while still immersed to midchest, and then returned to complete the WI. For HC trials, each subject entered the HC, was pressed to 1.35 ATA, and donned a hood supplying 100% O_2_ (PO_2_ = 1.35 atm). After the third hour, each subject removed his hood for a 10‐min lunch break while remaining at 1.35 ATA. After surfacing at the end of the trial, each subject emptied his bladder into a container; this post‐WI/HC volume plus that collected via his condom catheter (WI) or container (HC) were taken as his total urine output for the exposure. A final weight was taken after post‐WI/HC urination when the subject had dried completely. The difference in pre‐ and post‐WI/HC weights represents the weight lost during the exposure.

### Protocol for pre‐ and posttrial testing

Subjects lay supine on a tilt table with their arms outstretched; the tilt table (model 9505‐345; Bailey Manufacturing Company, Lodi, OH) was modified to support a person's arms at the level of his heart when he was tilted up. Subjects were then instrumented for measurement of HR (electrocardiogram), BP (Finometer Pro; Finapres Medical Systems, Amsterdam‐Zuidoost, the Netherlands), and limb blood flow (venous occlusion plethysmography). $\dot{Q}$ was obtained from a Finometer or by echocardiography, as described in Hemodynamic measurements. After instrumentation and a 15‐min supine adaptation period, a venous blood sample was obtained from the left antecubital vein for analysis of glucose, NE, aldosterone, atrial natriuretic peptide (ANP), AVP, hemoglobin (Hb), and hematocrit (Hct). This sample was taken approximately 30 min before HUT baseline measurements. Hemodynamic measurements were then taken at rest approximately 50 min after WI or HC exposure and during HUT approximately 1 h after WI or HC exposure. Pretilt data were recorded for 5 min following a physiological stabilization period after cold pressor and static handgrip tests, described elsewhere (Florian et al. 2013). Each subject was then tilted 70° head‐up from supine for 15 min or until symptoms associated with presyncope occurred or the subject requested termination of the test. Presyncope was defined as a rapid decrease in a systolic pressure to below 80 mmHg or a sustained systolic pressure below 90 mmHg associated with symptoms of light‐headedness, nausea, or diaphoresis. Subjects were tilted back down to the horizontal position at the end of 15 min or, if presyncope occurred, to the Trendelenburg position (−10°) until hemodynamic stability was reached. Ten minutes of recovery were recorded in the supine position. Tilt time was limited to 15 min due to schedule constraints. Only data segments from periods of hemodynamic stability (i.e., excluding presyncope and transients just after HUT) were analyzed.

### Water immersion

Twenty participants underwent 6‐h WI at the bottom of a 15‐ft pool filled with thermoneutral water (32–33°C). As noted previously, thermoneutral conditions varied between the individuals (Conaty et al. 2015; Pendergast et al. 2015). Since the water temperature provided in this study was optimized for subject comfort and is close to the generally accepted “thermoneutral” range, it will be referred to as “thermoneutral.” Ten of these subjects breathed humidified 100% O_2_ for an oxygen partial pressure (PO_2_) of approximately 1.35 atm (135 kPa), and the other 10 breathed humidified air for a PO_2_ of approximately 0.3 atm (30 kPa). They wore T‐shirts and shorts, and weights were provided to maintain negative buoyancy. While sitting upright in a chair, thus with the chest at a depth of approximately 12 ft, each participant breathed surfaced‐supplied O_2_ or air delivered with a MK20 breathing apparatus (Aga mask; Interspiro Inc., Pleasant Prairie, WI) (Florian et al. 2013). After 3 h of WI, each subject returned to the surface to stand on a platform with head and shoulders out of the water for 10 min while consuming a small lunch consisting of 2.2 MJ energy content (24% fat, 64% carbohydrate, 12% protein) and 500 mL total of a meal replacement and a sports drink.

### Hyperbaric chamber

Ten participants underwent a 6‐h HC trial in one of the dry chambers of the Navy Experimental Diving Unit Ocean Simulation Facility. Subjects wearing T‐shirts and shorts sat upright on a bench in the HC and were pressed to an equivalent pressure to the WI, that is, 1.35 ATA. Once the HC reached the appropriate depth, subjects donned oxygen treatment hoods (Amron Model 8891; Amron International, Vista, CA) and started their 6 h of breathing humidified 100% oxygen. After 3 h, subjects removed their hoods for 10 min while at depth and ate a lunch identical to that provided during WI exposures. Absolute pressure was kept constant for all phases so that the effects of WI and differences between hyperoxia and approximate normoxia (that is, with a slightly elevated PO_2_) on physiological function could be determined.

### Hemodynamic measurements

Resting brachial BP was measured oscillometrically (HEM 007XL; Omron Healthcare, Inc., Lake Forest, IL). Continuous BP was measured by photoplethysmography (Finometer) on a finger of the right hand. Finger pressure was calibrated to brachial artery pressure using the manufacturer's return‐to‐flow system. A 5‐lead surface electrocardiogram (Dash 3000; General Electric Company, Fairfield, CT) was used to determine HR.

#### Forearm blood flow

Forearm blood flow (FBF) was measured using venous occlusion plethysmography (model EC‐6; D.E. Hokanson, Inc., Malvern, PA). Data were recorded at baseline, every 5 min during HUT, and during recovery. During each data recording period, blood flow was acquired from three to four measurement cycles in succession. Forearm vascular resistance (FVR) was estimated as corresponding brachial MAP/FBF.

#### Cardiac output

$\dot{Q}$ was assessed using transthoracic echocardiography (Acuson Cypress; Siemens Medical Solutions USA Inc., Malvern, PA). Stroke volume (SV) was determined from the flow velocity across the aortic valve (apical approach) and the diameter of the aortic orifice during systole (parasternal long axis). $\dot{Q}$ was calculated as SV·HR and expressed in L/min. Systemic vascular resistance (SVR) was calculated as MAP/$\dot{Q}$. Orthostatic tolerance was estimated by the maximum increase in HR (+ΔHR_max_) during HUT and by the orthostatic index (Stegemann et al. 1975; Florian et al. 2013) calculated from the change in HR and BP during HUT.

### Time‐domain analyses and complexity analysis of HRV

#### Time‐domain HRV and BPV

Mean HR, root‐mean square of successive differences (RMSSD) of RR intervals, and the standard deviation of normal‐to‐normal R waves (SDNN) were calculated. RMSSD mainly reflects the modulation of the parasympathetic system and SDNN is an indicator of overall ANS activity. BPV was assessed using the standard deviation of mean SBP (SBP‐SD) (1996).

#### Approximate entropy (ApEn)

ApEn, a nonlinear statistical method used to assess the complexity of data, has been used to measure the loss of complex nonlinear HRV in a variety of pathological and physiological conditions (Pincus and Goldberger 1994; Florian et al. 2013). ApEn values were calculated from consecutive RR intervals using embedding dimension *m* = 2 and automatically selected threshold value *r* (Chon et al. 2009).

### Frequency‐domain analyses

A time‐domain HRV signal was generated from the instantaneous RR interval series at a uniform sampling rate of 4 Hz using cubic spline interpolation. The HRV signal was down‐sampled to 2 Hz, mean and linear trends were removed, and the signal was transformed into the frequency domain. For each subject, HRV time‐domain signal segments containing 360 points (3 min) were analyzed with both power spectral density (PSD) (1996) and principal dynamic modes (PDM) (Zhong et al. 2004) methods.

#### Power spectral density analysis of HRV

Power spectral densitys of HRV data were obtained using the Welch periodogram method (Matlab^®^ 7.9, Natick, MA). A 512 point Fast Fourier Transform (FFT), giving a frequency resolution of 0.004 Hz, was applied to data filtered with a 360‐point Hamming window and no overlapping segments. Mean spectral power in the low‐ (0.04–0.15 Hz) and high‐frequency (0.15–0.4 Hz) bands and the ratio between them were calculated (LF_HRV_, HF_HRV_, and LF/HF_HRV_, respectively). For LF_HRV_ and HF_HRV_, power was also represented normalized by total power (LFn_HRV_ and HFn_HRV_).

#### Principal dynamic modes (PDM) analysis of HRV

Principal dynamic modes analysis was used in addition to PSD to assess sympathetic and parasympathetic dynamics during HUT. Unlike PSD, PDM accounts for the inherent nonlinear dynamics of HR control. The two most dominant PDMs are considered to represent sympathetic (SNS) and parasympathetic (PNS) nervous activity. Methodological details have been described previously (Zhong et al. 2004). In this study, the optimal estimation error was found with nine Laguerre functions and a memory length of 60.

#### Baroreflex sensitivity

The complex‐valued transfer function between RR and SBP was evaluated as the ratio of the cross‐spectral density function of the two series and the PSD of the SBP series. The BRS gain (transfer function modulus) was determined by averaging the gain in the whole LF band (GainLF) regardless of the value of coherence (Pinna et al. 2002) within the LF.

### Blood samples

Glucose, Hb, and Hct levels were determined immediately after blood collection (Rapidpoint 400; Siemens Medical Solutions USA Inc). Blood for all other analyses was centrifuged at 4°C and stored at −80°C until assay. Samples for norepinephrine, ANP, AVP, and 8‐isoprostane were drawn into prechilled tubes containing EDTA. Blood for aldosterone was allowed to clot at room temperature for 30 min before centrifugation. Glucose was measured by the oxidase method; norepinephrine by HPLC; and aldosterone, ANP, AVP, and 8‐isoprostane by immunoassay.

For calculation of PV, blood was drawn in a 2‐mL sodium heparin tube for measurements of Hb and Hct using Rapidpoint 400 (Siemens Medical Solutions USA Inc.). The relative change in PV (ΔPV) following WI was calculated from changes in Hb and Hct concentrations according to the Harrison modification of the Dill and Costill equation (Harrison et al. 1982).

### Statistical analysis

A mixed model repeated measures analysis of variance (ANOVA) was used to determine the effect of WI and hyperbaric oxygen on neural, hormonal, and hemodynamic variables. The within‐subject factors were pre/post and time, and the between‐subject factor was exposure, or phase. When appropriate, differences between factors were identified using the Bonferroni‐Holm correction. All statistical analyses were performed using SAS 9.2 (SAS Institute, Inc., Cary, NC). The level of significance was set at *α *= 0.05, and values are presented as mean ± SEM. When significant, *P*‐values for main effects and interactions are noted on the figures.

## Results

Table 2 shows mean weight loss after each exposure, adjusted for food and fluid intake during the exposure, as well as at 0–3 h and at 3–6 h, total urine production, and and ΔPV after each exposure. Weight loss, ΔPV, and 0–3 h and total urine production were greater for Air and O_2_ WI compared with O_2_ HC (*P* < 0.05).

**Table 2:** Weight loss, urine production, and Δ plasma volume

	Air WI	O_2_ WI	O_2_ HC	*P*‐value
Weight loss, kg	−2.09 ± 0.09^a^	−1.78 ± 0.14^a^	−0.29 ± 0.12^b^	**<0.001**
3‐hour urine, mL	998 ± 116	775 ± 87	83 ± 59^b^	**<0.001**
6‐hour urine, mL	591 ± 70	590 ± 89	323 ± 41	0.568
Total urine, mL	1590 ± 131	1364 ± 146	406 ± 94^b^	**<0.001**
Δ Plasma volume, %	−11.3 ± 1.2^a^	−9.9 ± 2.1^a^	−1.3 ± 1.0^b^	**<0.001**

Values are mean ± SEM for urine production and postexposure weight loss and Δ plasma volume. Boldface values indicate statistical significance. Weight loss, 3‐h urine, total urine, and Δ plasma volume were significantly larger for Air WI and O_2_ WI compared with O_2_ HC.

WI, water immersion; HC, hyperbaric chamber.

a *P *< 0.01 compared to 0 changes.

b *P* < 0.001 compared to Air WI and O_2_ WI.

### Supine resting measurements

Supine pre‐ and postexposure hormone and electrolyte concentrations, as well as resting hemodynamic data, are displayed in Table 3. Due to technical issues, no AVP data are available for O_2_ HC exposures. No changes were noted for urine specific gravity, ANP, AVP, 8‐isoprostane, glucose, HR, and FVR for any of the phases. Significant phase × pre/postinteractions were identified for weight, NE, Hb, Hct, SBP, DBP, $\dot{Q}$, SV, and FBF. Specifically, in accord with the weight loss and decrease in PV following WI, Hb and Hct concentrations were also increased following WI only. SBP and FBF decreased after Air WI, but did not change after O_2_ WI, suggesting a compensatory response of hyperoxia to these postimmersion supine variations. Breathing O_2_ during WI also caused a decrease in $\dot{Q}$ and SV and an increase in NE and SVR that was not observed after Air WI. In contrast, DBP and FBF showed a significant increase after the O_2_ HC exposure that was not observed after O_2_ WI.

**Table 3:** Values of variables during supine rest before and after 6‐h water immersion or hyperbaric chamber exposure

	Air WI		O_2_ WI		O_2_ HC		Interaction
	Pre	Post	Pre	Post	Pre	Post	Phase × Pre/Post
Weight, kg	85.7 ± 2.3	84.3 ± 2.3**	88.6 ± 2.1	87.5 ± 2.1**	83.8 ± 2.3	83.8 ± 2.3	**<0.001**
Urine specific gravity	1.014 ± 0.002	1.012 ± 0.001	1.013 ± 0.003	1.010 ± 0.001	1.018 ± 0.001	1.012 ± 0.002	0.164
Aldosterone, ng/dL	8.37 ± 1.74	4.68 ± 1.47	5.10 ± 0.68	3.35 ± 0.69	9.25 ± 1.08	6.35 ± 1.00**	0.530
ANP, pg/mL	752 ± 131	706 ± 59	316 ± 100	299 ± 87	402 ± 49	345 ± 34	0.846
Arginine vasopressin, pg/mL	5.42 ± 1.03	5.48 ± 0.90	7.13 ± 1.89	7.79 ± 1.31	–	–	0.320
Norepinephrine, nmol/L	0.894 ± 0.114	1.151 ± 0.132	1.539 ± 0.183	2.429 ± 0.408*	0.906 ± 0.105	0.996 ± 0.155	**0.043**
8‐isoprostane, pg/mL	24.57 ± 1.86	22.26 ± 2.09	32.77 ± 8.52	38.31 ± 10.94	20.23 ± 2.29	18.48 ± 1.65	0.661
Glucose, mg/dL	94.3 ± 4.1	90.6 ± 2.1	93.0 ± 3.2	88.6 ± 2.3	83.8 ± 4.3	89.0 ± 1.6	0.984
Hemoglobin, g/dL	14.57 ± 0.30	15.54 ± 0.31**	14.54 ± 0.26	15.54 ± 0.19**	14.81 ± 0.20	14.86 ± 0.18	**<0.001**
Hematocrit, %	43.29 ± 0.88	46.39 ± 0.80**	42.26 ± 0.68	45.2 ± 0.64**	43.63 ± 0.63	43.67 ± 0.51	**<0.001**
Heart rate, beats/min	54 ± 3	51 ± 3	55 ± 2	54 ± 2	50 ± 2	50 ± 2	0.234
SBP, mmHg	131 ± 3	123 ± 3**	133 ± 3	133 ± 2	128 ± 3	134 ± 4	**0.003**
DBP, mmHg	73 ± 2	72 ± 2	74 ± 2	77 ± 2	71 ± 1	79 ± 2**	**0.003**
Cardiac output, l/min	4.8 ± 0.5	4.3 ± 0.3	5.0 ± 0.3	4.4 ± 0.3*	4.4 ± 0.2	4.5 ± 0.3	**0.036**
Stroke volume, mL/beat	91 ± 6	87 ± 7	94 ± 5	84 ± 4**	90 ± 3	94 ± 5	**0.005**
SVR, units	20.6 ± 1.6	21.4 ± 1.0	19.8 ± 1.4	21.7 ± 1.3*	20.8 ± 1.0	22.5 ± 1.7	0.659
FBF, mL/100 mL/min	4.35 ± 0.77	2.87 ± 0.44*	5.33 ± 0.83	4.43 ± 0.84	3.49 ± 0.47	5.10 ± 0.64*	**<0.001**
FVR, units	31.0 ± 7.8	36.6 ± 3.9	21.8 ± 3.5	28.3 ± 5.5	36.7 ± 8.6	24.2 ± 4.0	0.070

Values are mean ± SEM for pre‐ and postexposure variables for Air WI, O_2_ WI, and O_2_ HC. Boldface values indicate statistical significance. SBP, systolic blood pressure; DBP, diastolic blood pressure; SVR, systemic vascular resistance; FBF, forearm blood flow; FVR, forearm vascular resistance; WI, water immersion; HC, hyperbaric chamber. Interaction, *P*‐value of a two‐way (phase × pre/post) repeated measures ANOVA interaction.

*P < 0.05, **P < 0.01 compared with Pre.

### HUT testing

**Figure 1:**
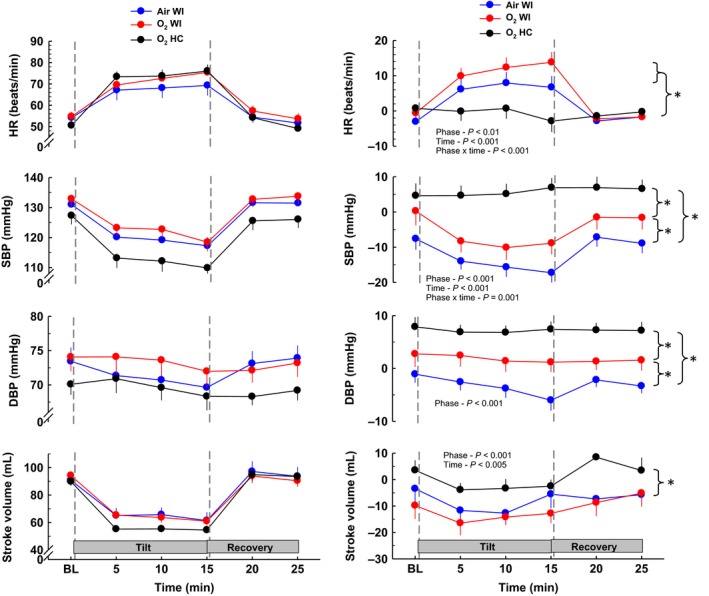
Hemodynamic responses before, during, and after 70° head‐up tilt testing. The left panel shows responses before 6‐h Air WI, O_2_ WI, and O_2_ HC exposure. The right panel shows the change in HUT responses after exposure (post‐minus‐preexposure). HR, heart rate; SBP, systolic blood pressure; DBP, diastolic blood pressure; WI, water immersion; HC, hyperbaric chamber. Values are group means ± SEM. Statistical comparisons between phases are noted on each plot. * denotes differences between phases. See Table 5 for pre/post directional responses and statistics.

**Figure 2:**
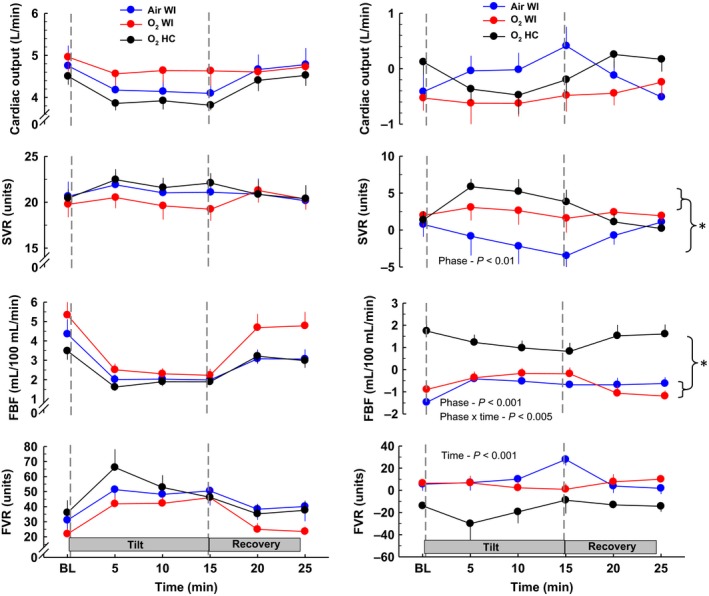
Blood flow and vascular resistance responses before, during, and after 70° head‐up tilt testing. The left panel shows responses before 6‐h Air WI, O_2_ WI, and O_2_ HC exposure. The right panel shows the change in HUT responses after exposure (post‐minus‐preexposure). SVR, systemic vascular resistance; FBF, forearm blood flow; FVR, forearm vascular resistance; WI, water immersion; HC, hyperbaric chamber. Values are group means ± SEM. Statistical comparisons between phases are noted on each plot. * denotes differences between phases. See Table 5 for pre/post directional responses and statistics.

**Figure 3:**
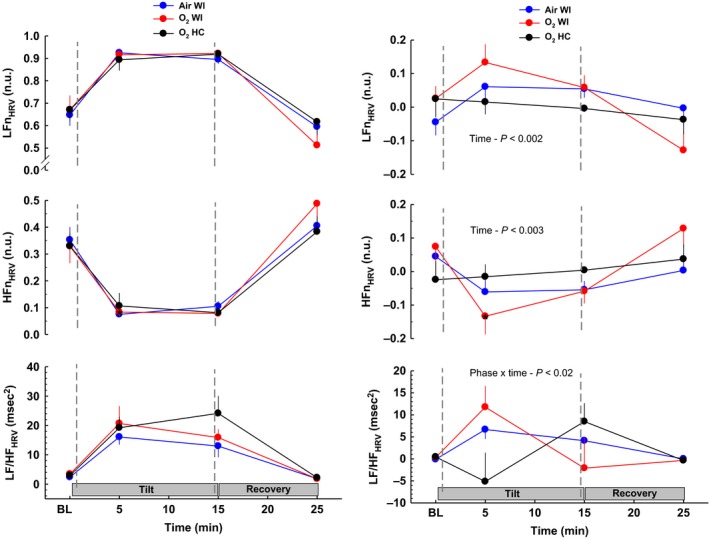
Heart rate variability responses before, during, and after 70° head‐up tilt testing. The left panel shows responses before 6‐h Air WI, O_2_ WI, and O_2_ HC exposure. The right panel shows the change in HUT responses after exposure (post‐minus‐preexposure). Heart rate variability did not change after O_2_ HC exposure. Data indicate a shift toward greater cardiac sympathetic dominance following WI. LF, low frequency; HF, high frequency; HRV, heart rate variability; WI, water immersion; HC, hyperbaric chamber. Values are group means ± SEM. Statistical comparisons between phases are noted on each plot. See Table 5 for pre/post directional responses and statistics.

**Figure 4:**
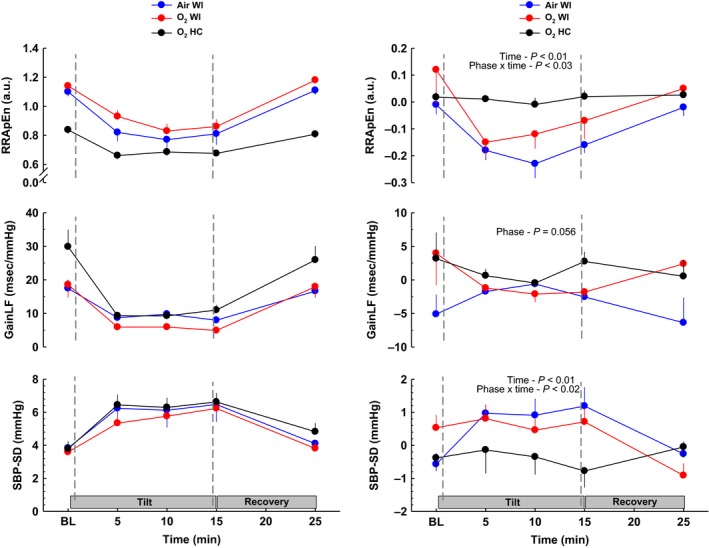
Complexity (RR ApEn), baroreflex sensitivity (GainLF), and blood pressure variability (SBP‐SD) responses before, during, and after 70° head‐up tilt testing. The left panel shows responses before 6‐h Air WI, O_2_ WI, and O_2_ HC exposure. The right panel shows the change in HUT responses after exposure (post‐minus‐preexposure). LF, low frequency; SBP‐SD, standard deviation of mean SBP; WI, water immersion; HC, hyperbaric chamber. Values are group means ± SEM. Statistical comparisons between phases are noted on each plot. See Table 5 for pre/post directional responses and statistics.

Divergent orthostatic responses (Tables 4, 5, Figs 1, 2, 3, 4) were evident for the three different exposures. Although all subjects completed HUT before Air WI, three subjects reached presyncope after Air WI exposure at 10.4, 9.4, and 6.9 min. One subject became presyncopal at 14.3 min before O_2_ WI and at 14.9 min after O_2_ WI exposure. One subject reached presyncope at 11 min before O_2_ HC but completed the full 15 min after O_2_ HC. Both −ΔHUT SBP_max_ and −ΔHUT DBP_max_ significantly increased after Air WI, and −Δ HUT SBP_max_ but not −Δ HUT DBP_max_ increased after O_2_ WI. Significant phase × pre/postinteractions were present for all orthostatic tolerance variables, including HUT time.

**Table 4:** Orthostatic responses before and after 6‐h water immersion or hyperbaric chamber exposure

	Air WI		O_2_ WI		O_2_ HC		Interaction
	Pre	Post	Pre	Post	Pre	Post	Phase × Pre/Post
Head‐up tilt time, min	15.0 ± 0.0	13.2 ± 1.0**	14.9 ± 0.1	15.0 ± 0.0	14.7 ± 0.3	15.0 ± 0.0	**0.022**
Orthostatic Index, units	52.1 ± 7.7	93.1 ± 12.9**	55.3 ± 4.2	110.6 ± 16.0**	86.1 ± 10.0	77.2 ± 8.5	**<0.001**
Δ tilt HR_max_, beats/min	31.1 ± 4.0	45.6 ± 5.1**	32.3 ± 1.6	48.3 ± 4.2**	42.9 ± 3.0	40.9 ± 3.3	**<0.001**
Δ tilt SBP_max_, mmHg	−28.9 ± 4.4	−38.2 ± 3.8**	−28.6 ± 2.8	−40.6 ± 4.5*	−35.2 ± 2.9	−31.5 ± 2.3	**0.018**
Δ tilt DBP_max_, mmHg	−15.3 ± 2.2	−18.5 ± 2.2*	−12.4 ± 1.9	−14.5 ± 2.0	−15.4 ± 1.8	−14.8 ± 1.1	**<0.001**

Values are mean ± SEM for pre‐ and postexposure variables for Air WI, O_2_ WI, and O_2_ HC. Boldface values indicate statistical significance. SBP, systolic blood pressure; DBP, diastolic blood pressure; WI, water immersion; HC, hyperbaric chamber, HR, heart rate. Interaction, *P*‐value of a two‐way (phase × pre/post) repeated measures ANOVA interaction.

*P < 0.05, **P < 0.01 compared with Pre.

**Table 5:** Comparisons of changes in variables during head‐up tilt before and after 6‐h water immersion or hyperbaric chamber exposure

	Air WI	O_2_ WI	O_2_ HC
Heart rate, beats/min	**↑** (+10.2%)	**↑↑** (+16.6%)	↔
Systolic blood pressure, mmHg	**↓↓** (−13.1%)	**↓↓** (−7.5%)	**↑↑** (+5.2%)
Diastolic blood pressure, mmHg	**↓↓** (−5.6%)	↔	**↑↑** (+10%)
Pulse pressure, mmHg	**↓** (−10%)	**↓↓** (−14.1%)	↔
Stroke volume, mL/beat	**↓↓** (−15.9%)	**↓↓** (−22.9%)	↔
Cardiac output, L/min	↔	**↓↓** (−12.5%)	**↓** (−7%)
Systemic vascular resistance, units	**↓** (−9.4%)	**↑↑** (+12.3%)	**↑↑** (+20.1%)
Forearm blood flow, mL/100 mL^/^min	**↓↓** (−26.2%)	↔	**↑↑** (+51.5%)
Forearm vascular resistance, units	**↑** (+27.6%)	↔	**↓↓** (−29.9%)
SDNN, ms	↔	↔	↔
RMSSD, ms	↔	**↓↓** (−29.6%)	↔
Transfer gain LF, ms/mmHg	**↓↓** (−16.6%)	**↓↓** (−24.1%)	↔
SBP‐SD, mmHg	**↑↑** (+20.3%)	**↑↑** (+13.2%)	↔
Approximate entropy, AU	**↓↓** (−18.4%)	**↓↓** (−11.2%)	↔
LFn, n.u.	**↑** (+6.9%)	**↑↑** (+11.7%)	↔
HFn, n.u.	**↓** (−37%)	**↓↓** (−53.5%)	↔
LF/HF, ms^2^	**↑** (+62.5%)	↔	↔
SNS	**↑** (+21.3%)	**↑** (+20.1%)	↔
PNS	↔	↔	↔
SNS/PNS	↔	↔	↔

Direction of change in effect of HUT on variables and associated *P*‐value during postexposure compared to preexposure head‐up tilt testing. A ↑ or ↓ is shown for *P*‐value <0.05, and ↑↑ or ↓↓ is shown for *P*‐value <0.01. Postexposure values show the divergent effects that water immersion (WI) and breathing O_2_ in a hyperbaric chamber (O_2_ HC) have on hemodynamic variables during head‐up tilt, and the combination of the two independent variables during O_2_ WI.

SDNN, standard deviation of normal‐to‐normal R waves; RMSSD, root‐mean square of successive differences of RRI; SBP‐SD, standard deviation of mean SBP; LF, low frequency; HF, high frequency; SNS, sympathetic nervous system; PNS, parasympathetic nervous system.

Hemodynamic and autonomic measurements before, during, and after HUT for all phases are presented in Tables 4, 5 and Figures 1, 2, 3, 4. Table 5 shows pre‐to‐postdive directional changes in the effects of HUT and % values when significant. A ↑ or ↓ is shown for *P*‐value <0.05, and ↑↑ or ↓↓ is shown for *P*‐value <0.01. HUT responses before and after Air WI have been documented previously (Florian et al. 2013), but pre‐Air WI HUT responses are included with O_2_ WI and O_2_ HC in the left panel of Figures 1, 2, 3, 4 for comparison purposes. The right panel shows the pre‐to‐post Δ (post‐minus‐pre) to highlight the effects that WI and/or hyperbaric O_2_ have on HUT responses. For the right panel, a positive or negative slope indicates a larger response during postexposure HUT; a straight line indicates no change in response (if centered at 0) or a larger response that occurs throughout baseline, HUT, and recovery (if positive or negative deviation from 0). Statistical comparisons among the postexposure HUT changes are shown.

#### Hemodynamic measurements

Water immersion caused changes in the HUT values of HR, SBP, PP, and SV, changes that were not compensated by breathing O_2_. HR during HUT was greater after than before exposure (+10 and +17%), while SBP (−13 and −8%), PP (−10 and −14%), and SV (−16 and −23%) during HUT were lower after exposure to Air WI and O_2_ WI, respectively. These changes seem related only to the WI; HR, PP, and SV during HUT were not different after O_2_ HC, while SBP and DBP during HUT were greater (+5 and +10%) after than before O_2_ HC. On the other hand, hyperoxia induced different responses in the HUT values of $\dot{Q}$ and SVR. $\dot{Q}$ during HUT was unchanged from the preimmersion value after Air WI but was lower after than before exposures to both O_2_ WI (−13%) and O_2_ HC (−7%). Similarly, SVR during HUT was lower after than before exposure to Air WI (−9%) but higher after than before exposures to both O_2_ WI (+12%) and O_2_ HC (+20%). DBP during HUT was lower (−6%) after Air WI than preexposure, was unchanged relative to preexposure after O_2_ WI, and was greater (+10%) after O_2_ HC than before the exposure. Different patterns were also evident for the HUT values of FBF, which was lower (−26%) after than before Air WI, was unchanged relative to preimmersion after O_2_ WI, and was greater (+52%) after than before O_2_ HC, and of FVR, which, in an inverse pattern to that seen for FBF, was greater (+28%) after than before Air WI, was unchanged relative to preexposure after O_2_ WI, and was lower (−30%) after than before O_2_ HC. In Figures 1 and 2, a general pattern can be seen: Air WI and O_2_ HC are the two extremes of pre‐to‐post Δ values, and O_2_ WI falls between them.

When the pre‐to‐postexposure Δ values during HUT were compared for all exposures (phases), the main effect of phase was significant for DBP, SV, and SVR, and both the main effect of phase and the phase × time interaction were significant for HR, SBP, and FBF. Overall, the orthostatic variables and hemodynamic responses to HUT suggest an overall reduction in orthostatic tolerance after Air WI, a blunted reduction in orthostatic tolerance after O_2_ WI, and improved orthostatic tolerance after O_2_ HC.

#### Heart rate variability and approximate entropy

HUT LFn_HRV_ and HFn_HRV_ increased and decreased, respectively, following WI, but did not change after O_2_ HC. However, the pre‐ to postexposure Δ was not significantly different among phases. LF/HF increased only after Air WI, and RMSSD decreased only after O_2_ WI. Compared to pre‐WI exposures, SNS increased after the both WI exposures, but not after O_2_ HC, and PNS and SNS/PNS were not different after any exposure. The expected decrease in ApEn during HUT was larger after than before WI exposures, but the pre‐to‐post ApEn Δ during HUT did not change after O_2_ HC. The differing responses between phases (i.e., WI vs. HC) was also evident as a significant phase × time interaction (*P* < 0.03).

#### Baroreflex sensitivity and blood pressure variability

As expected, preexposure GainLF for all phases decreased during the beginning of HUT, plateaued through the rest of HUT, and returned to baseline values during recovery. Postexposure GainLF for Air WI and O_2_ WI decreased further than it had preexposure, but the change after exposure was not significantly different among phases. Compared to preexposure values, postexposure SBP‐SD increased significantly during HUT after Air WI and O_2_ WI, but did not change following O_2_ HC.

## Discussion

This study demonstrates divergent effects of WI, breathing 100% oxygen in a HC, and the combination of WI and hyperoxia on hemodynamic responses and HRV during supine rest and 70° HUT. The novel finding is that breathing hyperbaric oxygen during a 6‐h WI improves postimmersion cardiovascular compensatory responses to orthostatic stress. This protection persisted for at least 1.5 h after egress from the water and was associated with higher SBP, DBP, and SVR at rest and during HUT compared with responses after Air WI. The change in hemodynamic responses after hyperbaric oxygen exposure was not accompanied by any changes in HRV or HR complexity measures. Lastly, after O_2_ HC exposure, although SVR increased during HUT, FVR decreased, indicating that constriction was not a global phenomenon even though most vascular beds must have been constricted to produce the whole‐body increase in SVR. Although the underlying mechanisms cannot be determined from the current study, these results suggest a robust and persistent vasoconstriction related to hyperbaric oxygen.

### Baseline responses

As expected and in agreement with previous studies (Mourot et al. 2004; Boussuges et al. 2009; Florian et al. 2013), weight decreased and PV was diminished by 9–11% after WI exposures but not after O_2_ HC exposure. In concord, 3‐h and total urine production were higher after WI exposures than after O_2_ HC exposure. In agreement with previous studies (Epstein 1992; Stadeager et al. 1992; Florian et al. 2013), aldosterone, ANP, and AVP were unchanged 1 h after immersion, independent of breathing gas. However, during supine rest NE was elevated after O_2_ WI but not after Air WI or O_2_ HC, suggesting that the combination of hyperoxic exposure and direct or indirect effects of WI are required to mount a sympathetic response. In contrast to the reductions in SBP and FBF and increase in CVR after Air WI, after O_2_ WI, SBP and FBF were preserved and SVR significantly increased. Interestingly, after O_2_ HC, FBF was increased even with an increase in DBP and trend toward increased SVR and reduced FVR, which suggests that other non‐muscular vascular beds may be constricted postexposure to compensate for muscle vasodilation. Similar to information previously reported for Air WI (Florian et al. 2013), subjects after exposures to O_2_ WI and O_2_ HC did not experience a change in resting supine HR and HRV. In spite of the noted vascular effects, it is possible that cardiac autonomic compensation is not necessary for supine individuals with mild hypovolemia (Kimmerly and Shoemaker 2002) or with residual vascular effects of hyperoxia as seen in this study.

### Head‐up tilt responses and WI

Individuals who maintained adequate arterial pressure during an orthostatic challenge typically mount an appropriate cardiac and arterial vasoconstrictor response to the gravitational blood translocation (Blomqvist 1986). However, post‐WI hypovolemia and altered autonomic regulation predispose individuals to orthostatic intolerance. Indeed, orthostatic intolerance has been repeatedly observed after long‐duration WI (Stegemann et al. 1975; Faes et al. 2013; Florian et al. 2013) and 3 days of dry immersion (Iwase et al. 2000). Results from this study and others (Faes et al. 2013; Florian et al. 2013) suggest that in addition to diminished blood volume, changes in cardiac function, reduced baroreflex sensitivity and coupling, and decreased vascular responsiveness, are responsible for the reduced orthostatic tolerance. However, the clear differences among phases in this study show divergent physiological effects of WI and hyperoxia on cardiovascular function, and in particular, a possible protective effect of hyperoxia on orthostatic tolerance, as evidenced by HUT time and hemodynamic responses during HUT.

### Oxygen effects

Normobaric and hyperbaric hyperoxia increased vascular resistance and alter cardiac and baroreflex regulation in patients, healthy participants, and animals. The increased vascular constriction has been shown to be nonsympathetically mediated (Seals and Victor 1991) but is induced rather by changes in reactive O_2_ species levels (Jackson 1987), endothelin‐1 (Dallinger et al. 2000), prostaglandins (Rousseau et al. 2010), or endothelium‐derived vasoactive products (Pasgaard et al. 2007). It is unclear to what extent the constriction is attributed to endothelium‐dependent vasoconstriction versus an attenuated endothelium‐derived vasorelaxation, but recent evidence suggests that reduced release and availability of nitric oxide (NO) plays a major role in the hyperoxic vasoconstriction (Pasgaard et al. 2007). Hyperoxia also reduces $\dot{Q}$ via baroreflex‐mediated vagal activation and diminished cardiac contractility (Demchenko et al. 2013). There is a linear increase in arterial–cardiac baroreflex function as the fraction of inspired O_2_ increases to 100% under normobaric conditions (Shibata et al. 2005), but the cardiac baroreflex slope has also been shown to decrease during hyperbaric hyperoxia (Gole et al. 2011). Nevertheless, the hyperoxic bradycardia is regulated by an increase and decrease in cardiac parasympathetic and sympathetic activity, respectively, as evidenced by changes in HRV (Lund et al. 1999, 2000, 2003; Shibata et al. 2005; Gole et al. 2011; Florian et al. 2013).

Although baseline and HUT testing in the current study was completed approximately 1 h postexposure, many of the aforementioned hyperoxic responses were still present. Compared to preexposure HUT testing, post‐O_2_ HC SBP, DBP, and SVR remained elevated and $\dot{Q}$ was depressed throughout HUT testing. The hyperoxic constrictive effects also carried over to the post‐O_2_ WI HUT testing where, in contrast to the reduction in SVR and larger Δ tilt DBP_max_ after Air WI, SVR increased and Δ tilt DBP_max_ did not change. $\dot{Q}$ was also reduced across post‐O_2_ WI HUT testing.

Several studies have shown that cardiovascular adjustments to hyperoxia can persist after return to normoxia. For example, $\dot{Q}$ and HR did not return to baseline levels after 55 min of recovery following intermittent exposure to 100% O_2_ at 2.2 ATA (Pelaia et al. 1992). The reductions in $\dot{Q}$, SV, BRS, and LF_BPV_, and an increase in SVR were still evident 10 min after a 45‐min exposure to 100% O_2_ at 1 ATA, but returned to baseline levels by 30 min postexposure. The cardiac parasympathetic shift during the same hyperoxic exposure was more evanescent than the vascular responses, with a rapid postexposure shift to sympathetic dominance that lasted at least 30 min after return to normoxia (Gole et al. 2011). In the current study, no HRV or complexity measures differed from predive when they were measured 1 h postexposure after O_2_ HC, suggesting full or nearly full recovery from dry hyperoxia in that time. In contrast, 1 h after either immersed condition, LFn_HRV_ and SNS were increased and HFn_HRV_ decreased relative to baseline, and SBP‐SD was also higher, reflecting a persistent sympathetic dominance post‐WI independent of hyperoxia. The reduction in GainLF after WI likely reflects a deterioration of BRS. Together, these changes point toward increased cardiac sympathetic activation up to 1.5 h after WI independent of breathing gas, but hyperoxia may still override some of the changes from WI such as reduced SVR and BRS.

Interestingly, during HUT after O_2_ HC despite an increase in SVR, FBF was augmented and FVR diminished. This response contrasts with the reduced FBF and increased FVR after Air WI, and no change in either variable after O_2_ WI. The observation that after O_2_ HC BP and SVR were elevated while $\dot{Q}$ was slightly reduced and FBF was increased suggests a redistribution of blood flow and vasoconstriction in vascular beds other than muscle such as the splanchnic region. In animals, during short‐term hyperoxia, blood flow to the mesenteric circulation decreases, as, to a much lesser extent, does blood flow to muscle vasculature in the limbs (Bergofsky and Bertun 1966; Torbati et al. 1979). However, to our knowledge there are no data from which to extrapolate vascular recovery responses to the current posthyperoxic recovery data, nor are there any related experiments that have provided hyperbaric oxygen for as long a time. It is possible that the increase in FBF after O_2_ HC is a compensatory response similar to the posthyperoxia HRV shift (Gole et al. 2011) or rebound to the hyperoxic constriction. What is clear is that nonmuscle (or nonforearm muscle) constriction persisted long enough after cessation of hyperoxia to increase post‐O_2_ HC BP during HUT and to maintain BP during HUT after O_2_ WI.

### Experimental considerations

Several factors should be considered when interpreting the results of this study. Supine and HUT measurements were taken approximately 1–1.5 h postexposure, respectively, so the timeframe for manifestation of residual/compensatory physiological factors before that time cannot be determined. Similarly, since all exposures in this study were for 6 h, the duration or dose of oxygen required to provide protective effects cannot be determined from the current study. For comparison to previous studies, it is important to note that there may be differences between head‐in WI and head‐out WI (Pendergast et al. 2015). However, the seated head‐in WI with a demand regulator at the mouth used in our experiments provides the same pulmonary hydrostatic loading as does seated head‐out WI. Although most studies conduct HUT tests to failure, due to diving operation constraints, HUT was limited to a maximum tilt time of 15 min. Therefore, in addition to analyzing HUT time, other measures of orthostatic tolerance and hemodynamic stability such as the orthostatic index and changes in BP and HR during HUT were analyzed. An additional consideration is the use of echocardiography to measure Q. This indirect method provides reliable relative changes but may underestimate absolute values (Kiowski et al. 1981). Because HRV is an indirect measure of cardiac sympathetic and parasympathetic activity, it may not always accurately indicate sympathetic activation of the heart or be generalizable to other regions. However, LFn_HRV_ and LF_BPV_ responses during HUT have been shown to parallel changes in peroneal MSNA (Furlan et al. 2000). Lastly, depending on the PO_2_ and duration of exposure, hyperoxia may cause central nervous system (CNS) oxygen toxicity and pulmonary oxygen toxicity. However, military and technical divers commonly use the PO_2_ employed in this study because it is low enough that CNS oxygen toxicity is not a concern. Indeed, none of the subjects in the current study experienced symptoms of CNS oxygen toxicity, and we measured a generally mild pulmonary oxygen toxicity effect which is reported elsewhere.

## Conclusion

This study demonstrates the divergent effects that WI, breathing 100% oxygen in a HC, and the combination of WI and hyperoxia have on hemodynamic responses and HRV during supine rest and 70° HUT. Breathing hyperbaric oxygen during a 6‐h WI augments postimmersion cardiovascular compensatory responses to orthostatic stress. The primary protective effect appears to emanate from increased constriction in nonmuscle vasculature. Future mechanistic studies are required to determine both the timeline for hyperoxia residual/compensatory effects and which vascular beds are responsible for maintaining and even increasing SVR.

## Conflict of interest

None declared.
